# Potential of the Other Genetic Information Coded by the Viral RNA Genomes as Antiviral Target

**DOI:** 10.3390/ph12010038

**Published:** 2019-03-13

**Authors:** Alfredo Berzal-Herranz, Cristina Romero-López, Beatriz Berzal-Herranz, Sara Ramos-Lorente

**Affiliations:** Instituto de Parasitología y Biomedicina López-Neyra, (IPBLN-CSIC); Av. del Conocimiento 17, PTS Granada, Armilla, 18016 Granada, Spain; cristina_romero@ipb.csic.es (C.R.-L.); bbh@ipb.csic.es (B.B.-H.); seramos@correo.ugr.es (S.R.-L.)

**Keywords:** Viral RNA genome, Functional RNA domains, RNA–RNA interactions, Antivirals, RNA aptamers, RNA structure/function, RNA tools

## Abstract

In addition to the protein coding information, viral RNA genomes code functional information in structurally conserved units termed functional RNA domains. These RNA domains play essential roles in the viral cycle (e.g., replication and translation). Understanding the molecular mechanisms behind their function is essential to understanding the viral infective cycle. Further, interfering with the function of the genomic RNA domains offers a potential means of developing antiviral strategies. Aptamers are good candidates for targeting structural RNA domains. Besides its potential as therapeutics, aptamers also provide an excellent tool for investigating the functionality of RNA domains in viral genomes. This review briefly summarizes the work carried out in our laboratory aimed at the structural and functional characterization of the hepatitis C virus (HCV) genomic RNA domains. It also describes the efforts we carried out for the development of antiviral aptamers targeting specific genomic domains of the HCV and the human immunodeficiency virus type-1 (HIV-1).

## 1. Introduction

The biological function of RNA has, for long time, been considered to be restricted to its role in the transmission of genetic information from DNA to proteins. The discovery in the early 1980′s that the RNA is capable of catalyzing chemical reactions [1,2] led to the reconsideration of this dogma and aroused the interest in unraveling the molecular mechanisms of the catalytic activity of RNA molecules and, later, in deciphering the unknown functional roles of RNA within cells. Currently, it is widely accepted that RNA plays a central role in many biological processes in all living organisms. An increasing number of functional RNA molecules, so-called “noncoding RNAs”, has been identified. Although they do not encode proteins, they encode information that is also essential for life. In fact, errors in RNA metabolism or the loss of function of RNA molecules are the cause of pathological processes of sanitary importance. Therefore, determining the function of certain RNA molecules has made it possible to clarify the molecular mechanisms of different biological processes. The genomes of RNA viruses constitute a particular class of functional RNA molecules. Viral RNA genomes are compact entities that carry all the information that the virus requires to complete the infectious cycle. Besides acting as a replication and translation template, viral RNA genomes play several essential functions for the completion of the viral cycle and their regulation. To achieve all these functions, they have developed an information coding system that complements and overlaps the protein coding one. This information coding system uses discrete RNA units that fold into their specific structures for storing information. These structural units are, in fact, functional genomic RNA domains and are highly conserved among the viral population. They can be grouped in complex folded RNA regions that interact among them to achieve the proper functioning. Thus, the structure of the entire genome has to be preserved for the efficient viral propagation. This structural conservation contrasts with the great sequence variability of the viral RNA genomes, which helps the viral populations to adapt to novel molecular and cellular contexts and to escape host defenses. It also contributes toward the development of resistance to antiviral drugs. The genome variability is, therefore, limited by the preservation of the genome structure. The precise equilibrium between the sequence variability and structure preservation determines the success of the RNA viral populations.

Deciphering the mechanisms that underlie each of the functions performed by functional genomic RNA domains and understanding the molecular strategies used by RNA genomes to contain all the information necessary for each of them are scientific problems of great relevance and interest. Elucidating these mechanisms would provide information of enormous importance to address the control of infections caused by RNA viruses. The current information indicates that structural RNA domains play out their different biological roles (e.g., in replication, translation, or encapsidation) by directly recruiting viral and/or cellular factors and/or by forming high-order structures via the establishment of long-range RNA–RNA interaction networks (for a review, see Reference [3]).

The essentiality of these genomic RNA domains for the virus makes them excellent candidates as targets for the development of antiviral strategies. Drugs aimed at interfering with their function either by blocking their interactions with viral or cellular factors or by impeding their proper folding may seriously compromise the viral viability. Therefore, they should be considered promising antiviral agents. Aptamers specifically recognize and efficiently bind to structural elements within a target molecule, being among the most promising molecules for interfering with the function of structural domains of viral RNA genomes.

Members of the family *Flaviviridae* bear a positive single-stranded RNA genome. They are responsible of worldwide public health problems. The family comprises three genera: *Flavivirus*, that includes important human pathogens like Dengue virus (DENV), West Nile virus (WNV), yellow fever virus (YFV), or Zika virus (ZIKV), among many others; *Pestivirus*, represented by the virus responsible of the Bovine viral diarrhea (BVDV) or the classic swine fever (CSFV); and *Hepacivirus*, with the hepatitis C virus (HCV) as the most significant member. Their genome contains multiple highly conserved structural RNA domains, which store essential information for the completion of the viral cycle. This review focuses on the HCV genome, paying special attention to the work carried out in our laboratory aimed at the structural and functional characterization of genomic RNA domains. The second part of the review summarizes the main strategies we have developed seeking aptamers targeted against specific genomic RNA domains of the HCV and HIV genomes. 

## 2. HCV Genomic Functional RNA Domains

**Figure 1:**
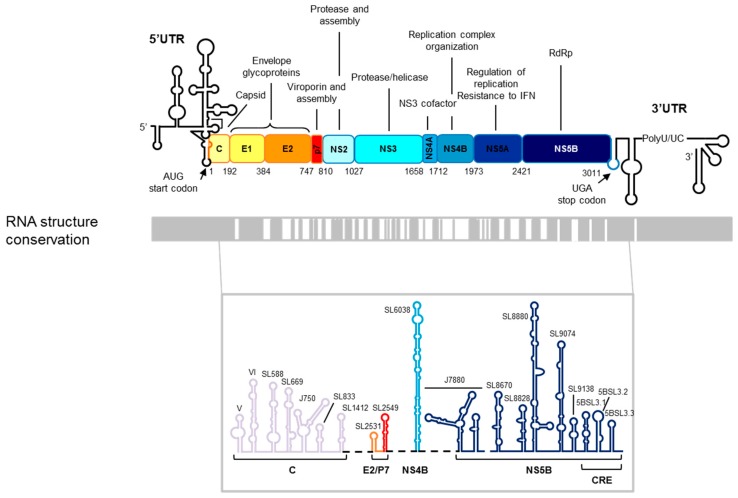
The hepatitis C virus (HCV) RNA genome. Upper panel: A schematic representation of the genetic organization of the viral genome. The 5′ and 3′ UTRs flanking the single ORF are depicted by a black line. The viral proteins and their functions are indicated. The translational start and stop codons are marked by arrows. The numbering corresponds to the codon positions in the ORF according to the HCV Con1 isolate, genotype 1b. Lower panel: A structural conservation map of the HCV RNA is represented by gray boxes denoting structurally conserved regions among different viral isolates. The predicted secondary structures of conserved elements in the ORF of the viral RNA genome are shown at the bottom. The color code and labels at the bottom indicate the position where each stem-loop is located. The figure is adapted from Reference [27].

HCV is likely the member of the family *Flaviviridae* that has attracted major efforts to understand the molecular mechanisms that govern its infective cycle. Its genome is a 9600-nt-long single-stranded RNA molecule, which codes for a single open reading frame (ORF) flanked by highly conserved untranslated regions (UTRs). Although the UTRs comprise numerous conserved structural/functional RNA elements that play essential roles for the consecution of the viral cycle, these RNA elements are also distributed throughout the coding region (Figure 1). The 5′ UTR is a highly conserved and complexly folded region that is mainly occupied by an internal ribosome entry site (IRES) that spans around 30 nt within the viral capsid coding region [4,5]. The IRES directs the recruitment of the 40S ribosomal subunit in the absence of a cap structure and initiates the viral protein synthesis [6,7]. It is folded into four structural domains that comprise a set of highly conserved subdomains, each with essential roles in the ribosome recruitment and translation initiation. In addition, 5′ UTR structural domains are essential for viral replication and infectivity [6,7,8,9,10,11,12]. The initiation of replication takes places at the 3′ UTR [8,10,11]. It is a highly conserved 200–250-nt-long region, which contains several functional RNA elements grouped into highly conserved domains required for efficient viral replication. The 3′ UTR also plays an important role in the viral translation, regulating the IRES activity [13,14]. 

The use of complementary experimental approaches (bioinformatics tools, secondary structure mapping, and genetic strategies) has provided a good overview of the HCV genome folding [15,16,17,18,19,20,21,22,23]. It has allowed the identification of up to 20 highly conserved structural elements among different viral isolates throughout the ORF of the HCV genome (Figure 1). This high structural conservation within a genome with a high rate of sequence variability indicates that each structural unit codes important information for the efficiency of the virus infective cycle. In contrast to the genomic UTRs, information about the structure and function of the ORF structural elements is scarce. Among them, the so-called CRE, cis-replication element, is probably the one that has attracted more attention (Figure 1). The CRE is defined by three stable stem-loops known as 5BSL3.1, 5BSL3.2, and 5BSL3.3 located at the 3′ end of the protein coding region [24,25]. The central domain, 5BSL3.2, is absolutely indispensable for HCV propagation, acting as an essential element for viral replication. Further, it has been shown that CRE is a negative regulator of the HCV IRES-dependent translation [26].

## 3. Long-Range Genomic RNA–RNA Interactions

**Figure 2:**
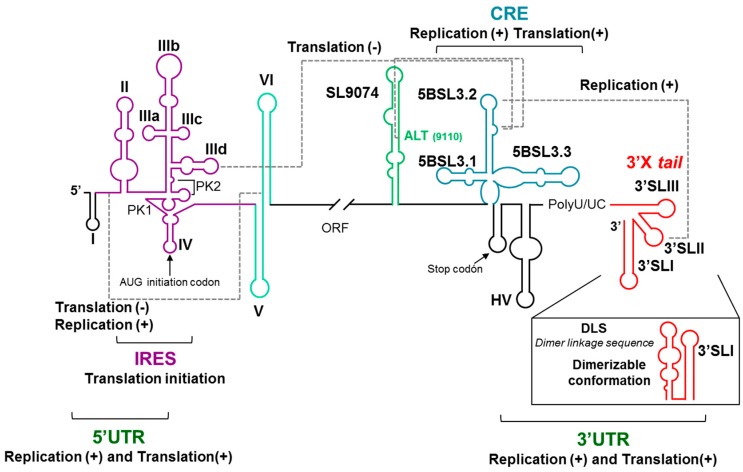
A schematic representation of the 5′ and 3′ ends of the HCV genome depicting the highly conserved structural RNA domains: The defined functional RNA–RNA interactions are indicated with broken gray lines. The translational start and stop codons are indicated by arrows. The figure is adapted from Reference [32].

Specific structural elements of the 5′ and 3′ ends of the HCV genome are involved in the viral replication and translation. This implies the existence of a communication between the two genomic ends. It was commonly accepted that the acquisition of a genomic circular conformation was mediated by the recruitment at both ends of the cellular and viral proteins, as it was demonstrated for the recruitment of IRES-activity stimulating proteins by the genomic HCV 3′ UTR [13,28,29,30]. We questioned whether the conserved structural RNA elements located in the two ends of the HCV genome were directly involved in the formation of the circular conformation. We first tested the ability of two independent RNA fragments comprising the 5′ or the 3′ genomic ends to bind with each other. Interestingly both RNA genomic ends form a stable complex in in vitro assays [31]. Further characterization allowed us to demonstrate that this interaction takes place between the two essential structural RNA elements IIId within domain III of the IRES and the 5BSL3.2 within the CRE (Figure 2). It responds to a kissing ALIL (apical loop–internal loop) interaction. This result represented the first evidence of a long-range RNA–RNA interaction involving the two genomic ends, supporting a protein-independent circularization of the HCV RNA genome. The functional characterization of this interaction allowed us to conclude that it actually occurs in vivo, being the CRE a negative regulator of the HCV IRES activity [26] (Figure 2). The use of CRE mutants depleted of specific structural units demonstrated that this translation regulation activity is mainly restricted to the central structural unit 5BSL3.2 [26].

The 5BSL3.2 domain is also involved in an Apical loop–Apical loop (ALAL) long-distance interaction with the highly conserved 3′SLII domain included in the 3′X tail region at the 3′ UTR [33,34] (Figure 2). This interaction promotes viral replication. A third functional long-range interaction has been described, which involves the internal loop of the 5BSL3.2 domain and the upstream Alt region [17,35,36] (Figure 2).

A functional RNA–RNA interaction seems to take place between domain VI, at the 5′ end of the protein coding region, and the highly conserved linker sequence between domains I and II within the 5′ UTR. This interaction has been proposed to participate in the regulation of both the viral replication and translation processes [20,37,38,39,40]. Interestingly, the interacting sequence within the interdomain region overlaps with the interaction site of the liver-specific micro RNA miR-122, which has been shown to be involved in viral replication and translation [41,42]. It has been proposed that the interaction of miR-122 would induce a conformational switch of the 5′ genomic end encompassing the IRES, which would be responsible for the regulation of the activity of this region [39].

All together, these results probe the existence of a network of long-range RNA–RNA interactions. This network involves essential structural genomic RNA domains of at least the IRES, CRE, and the genomic 3′ UTR X-tail region and governs the regulation of the essential viral processes (reviewed in References [27,43]; Figure 2), in which the CRE 5BSL3.2 domain seems to be the conductor of the orchestra directing the regulatory interactions of all other structural elements.

**Figure 3:**
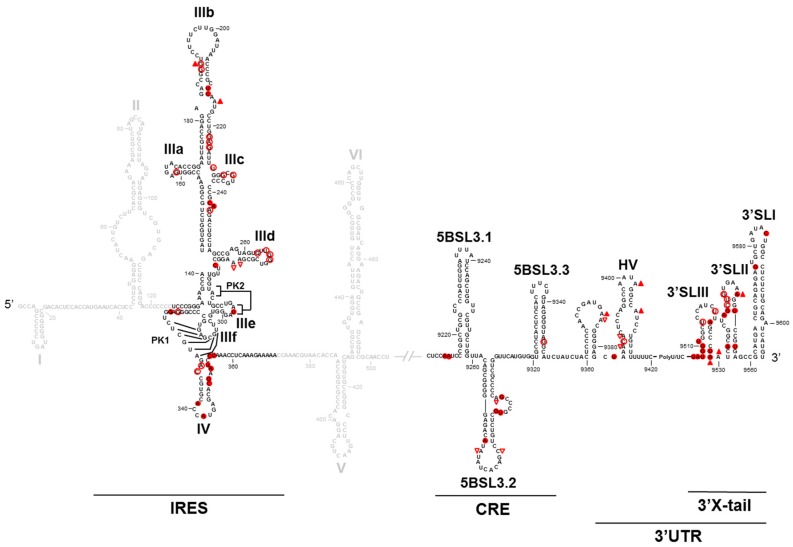
The conformational tuning of long-distance functional RNA domains: A summary of the comparative structural analysis of the 5′ and 3′ HCV genomic ends in the presence and absence of each other end. Only nucleotides that show a differential chemical reactivity are indicated with red figures. The solid figures indicate increases in reactivity. The empty figures indicate decreases in reactivity. △ indicates the DMS (dimethyl sulfate) reactivity. ○ indicates the NMIA (N-methyl-isatoic anhydride) reactivity.

To understand the molecular mechanism by which long-distance interactions may regulate viral processes, a detailed structural analysis at the nucleotide level of the HCV genome was carried out. For this purpose, a combination of chemical probing methods, SHAPE (Selective 2′-hydroxyl acylation and primer extension)-based technology analysis and accessibility using oligonucleotide microarrays, was used. The results of this analysis allowed us to conclude that interactions between elements of both ends of the viral genomes promote to each other their structural tuning at the secondary and tertiary levels, which undoubtedly leads to the mutual modulation of their functionality [32,44] (Figure 3). Each one tunes the structural conformation of essential domains of the other end of the genome. These structural modifications correlate well with the functional regulation observed. Thus, modulation of the translation efficiency at the 5′ genomic end by the 3′ end is achieved by promoting the conformational fine-tuning of the IRES-essential domains III and IV, while the 5′ genomic end promotes significant structural changes at the 3′ genomic end, mainly at the 3′X tail region. The latest could account for variations in the translational and replicational efficiencies. 

Besides intramolecular genomic RNA–RNA interactions, intermolecular ones have also been described. Thus, the dimerization of the HCV genome is initiated at a highly conserved palindromic sequence named DLS (Dimerization Linkage Sequence) located at the 3′SLII element of the 3′X tail region [45,46] (Figure 2). The 3′X tail region folds into two alternative conformations [46,47]: a three stem-loop (3′SLI-3′SLII-3′SLIII) [48] and a two stem-loop (3′SLI-3′SLII’) conformers, being the latest dimerization competent one, which exposes the DLS in the apical portion of the stem-loop SLII’. A functional analysis of the 3′X tail in the presence of the IRES and/or the CRE allowed us to demonstrate that genomic dimerization is modulated by long-distance elements [49]. This confirms the functionality of the described long-distance RNA–RNA interactions involving the CRE-3′X tail and IRES-3′X tail. 

All together, these data demonstrate the existence of a dynamic network of RNA–RNA contacts that tune the structural conformation of essential distal RNA elements, resulting in the regulation of the different stages of the viral cycle. Our data support that the 5BSL3.2 domain operates at the core of this regulatory system.

## 4. Functional Genomic RNA Domains as Therapeutic Targets

Interfering with the function of viral genomic structural domains either by modifying their folding or by impeding the interactions they are involved in offers a potential means to compromise their viral viability and in the consequence of fighting viral infections. Much effort has been devoted to the development of nucleic acid-based strategies aimed to interfere with the functioning of viral RNA genomes. Several types of RNA molecules have been shown to be efficient antiviral agents, and some of them have even entered clinical trials [50,51,52]. Out of the different inhibitor RNAs, aptamers have been postulated as promising tools for targeting structural RNA elements.

### 4.1. Aptamers

Aptamers are short single-stranded DNA or RNA oligonucleotides that recognize and bind efficiently and specifically to a target molecule [53,54]. Aptamers have been isolated against a wide range of molecules varying in nature, size, and complexity: from ions to full cells including small molecules (such as amino acids, nucleotides, antibiotics, or metabolites), peptides, proteins, nucleic acids, macromolecular aggregates, virus, cell organelles, or tissues (for examples, see References [55,56,57,58,59]). In all cases, aptamers are isolated following a common experimental in vitro strategy known as SELEX (Systematic Evolution of Ligands by EXponential enrichment) [54]. It consists in iterative selection cycles that comprise the following steps: binding to the target molecule, partitioning the binders, and molecularly amplifying the binders. The SELEX strategy allows the identification of efficient binder molecules from a large pool of variants, usually 10^12^–10^15^. The winner molecules are known as aptamers. Since it is an entirely in vitro process, the selection conditions are controlled by the researcher and can be modified along the process. 

Herein, we summarize the attempts performed at our laboratory to identify aptamers with a potential antiviral activity targeting highly conserved structural RNA elements within viral genomes, paying special attention to the work we have performed to develop RNA inhibitors targeting the HCV RNA genome. 

#### 4.1.1. Aptamers Targeting HCV IRES

**Figure 4:**
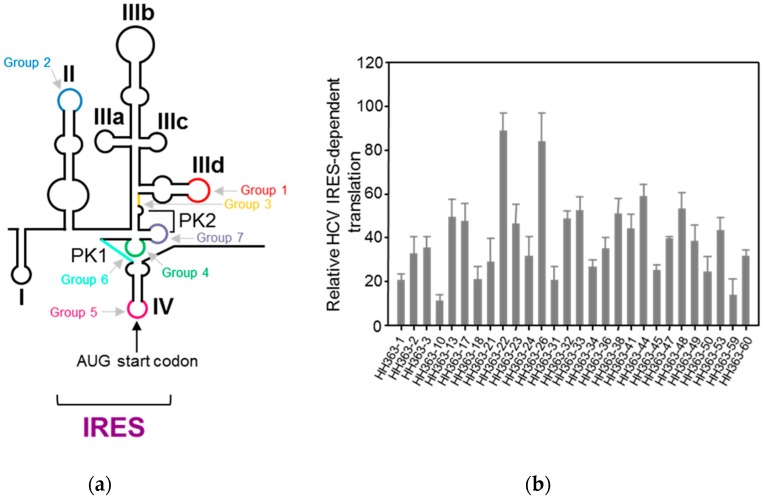
The selection of RNA inhibitors targeting the HCV internal ribosome entry site (IRES): (**a**) The identification of the aptamer targets within the IRES. The targets determined by the complementary sequence to the consensus motif that define each aptamer’s group are indicated using a colour code on the schematic representation of the secondary structure of the IRES. (**b**) The inhibition of the IRES activity by a collection of selected aptamers: The IRES-dependent translation was measured in vitro as the Fluc activity. The values are the mean of at least three independent experiments and are referred to the activity in the absence of an RNA inhibitor. Figure adapted from [61].

We developed an innovative SELEX strategy that was applied to a theoretical population of more than 1 × 10^15^ variant RNA molecules with lengths of 84 nt. The original population resulted from the randomization of 25 consecutive nucleotides linked at the 3′ end of the catalytically active hammerhead ribozyme targeted against position 363 of the HCV IRES. The resulting population was subjected to a two-sequential-selection-steps procedure [60]. The first selection step selected for the binding to the complexly folded HCV IRES, the aptamers’ selection; the second step selected for cleavage of the viral RNA by the ribozyme domain of the previously selected binders. After six rounds of selection, the heterogeneity of the population was analyzed and the resulting aptamers were classified within seven groups defined by a consensus sequence motif ranging from 5 to 9 nt in length. Each consensus motif is complementary to a specific sequence within the IRES (Figure 4a). This indicates that the complementary sequence partners are required for the binding of the two molecules. An in vitro analysis of the inhibition of the IRES activity indicated that the majority of the selected inhibitory RNAs significantly reduced the IRES-dependent translation, yielding inhibitions up to 90% [61] (Figure 4b).

**Figure 5:**
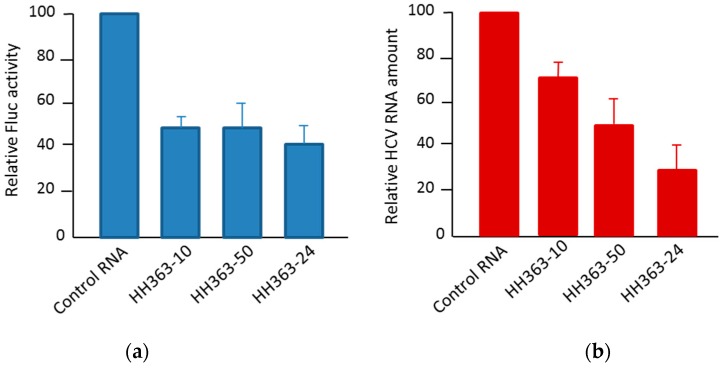
The antiviral activity of selected RNA inhibitors in a human hepatoma cell line. (**a**) The inhibition of HCV IRES-dependent translation in Huh-7 cells by three RNA inhibitors: The cells were co-transfected with a mix containing the transcripts IRES-Fluc and cap-RLuc mRNAs and an excess of a specific RNA inhibitor. The IRES translation efficiency was measured as Fluc versus RLuc activity and referred to the value obtained in the control reaction without an RNA inhibitor. The activity values are the mean of three independent experiments. (**b**) The inhibition of viral replication in a Huh-7 cell line-based HCV sub-genomic replication system (Huh-7 NS3-3′): The cells supporting the stable replication of an HCV sub-genomic replicon were transfected with individual RNA inhibitors and incubated for 20 h. The viral replication was measured as the viral RNA amount by qRT-PCR from a total cell RNA extraction. The data are the mean of three independent experiments.

A further in vivo analysis in the Huh-7 cell culture (human hepatoma cell line) of the most efficient in vitro inhibitors yielded efficient antiviral activities showing inhibitions up to 60% and 70% of the viral translation and replication, respectively [61,62,63,64] (Figure 5).

#### 4.1.2. Anti-HCV CRE Aptamers

The highly conserved genomic CRE element codes essential information for the regulation of the viral processes. It has been shown to be an essential partner in the viral replication [24,25], and it is involved in the translation regulation [26,65]. CRE is, therefore, an excellent candidate to be an efficient anti-HCV therapeutic target. To assess this hypothesis, we applied a SELEX procedure to isolate aptamers that could interfere with the functioning of the CRE element, which is defined by the information encoded by each of its structural domains (5BSL3.1-5BSL3.2 and 5BSL3.3).

**Figure 6:**
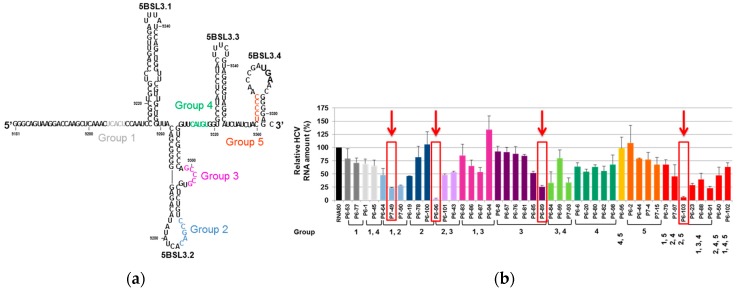
RNA aptamers selected against the HCV *cis*-replication element (CRE): (**a**) Sequence and secondary structure of the 194-nt-long viral RNA fragment used as the target for the selection of anti-HCV aptamers. The highly conserved structural domains of the CRE element (5BSL3.1, 5BSL3.2, and 5BSL3.3) and the 5BSL3.4 that includes the translational stop codon, are indicated. The target sequences complementary to the consensus sequence that define the five groups of selected aptamers are indicated by colored sequences. (**b**) The inhibition of HCV replication: Huh-7 cells supporting the stable replication of a sub-genomic HCV replicon were transfected with independent aptamers. Viral RNA levels were quantified from the total RNA extracted 18 h post-transfection using qRT-PCR. The bar chart indicates the HCV RNA levels referred to those obtained in the absence of a non-related RNA molecule, RNA80. The values are the mean of at least four independent experiments [67]. The red arrows and boxes indicate the aptamers for which the mechanism of action was further characterized.

For this assay, a starting population of RNA molecules resulting from the randomization of 30 contiguous nucleotides (yielding a theoretical heterogeneity of more than 1 × 10^18^ variants) was synthesized and introduced in a standard SELEX procedure. With this strategy, we searched for binders to a 194-nt-long viral genomic RNA fragment containing the CRE structural element [66] (Figure 6a). After nine rounds of selection, a collection of RNA aptamers could be classified in five different groups defined by a consensus sequence, which was complementary to a specific domain within the viral target RNA (Figure 6a). These complementary sequence motifs are likely involved in the binding of the two molecules. Interestingly, the majority of the selected aptamers contained more than one consensus sequence and potentially more than one site of binding in the target molecule [66,67]. To test the potential of the CRE as anti-HCV target, a collection of 44 aptamers was assayed for their capacity to inhibit HCV replication. For this purpose, the sub-genomic viral replication system based on the Huh-7 cell lines mentioned above (see Figure 5) was used. The results of this analysis showed than a significant number of tested aptamers efficiently reduced the viral RNA levels (Figure 6b). It is worth noting that those aptamers that yield the highest inhibition levels contained the consensus sequences of groups 2 and 3, which target the apical and internal loops of the 5BSL3.2 domain, respectively (Figure 6). Further characterization of the inhibitory effect of aptamers P6-89, P6-96, and P6-103 competed out the recruitment of the viral polymerase by the 5BSL3.2 region [67]. This result was also confirmed with the group 2 aptamers P-58 and P-78 [68]. Further, the P7-49 that is also an efficient HCV replication inhibitor seems to bind to the 5BSL3.4 rather than 5BSL3.2 domain. Our data suggests for the 5BSL3.4, which bears the translational stop codon, to have new unknown roles in the viral cycle.

Taken together, the results of this study support the potential use of the HCV CRE element as a therapeutic target. More importantly, they provide evidences that aptamers are also useful molecular tools for understanding the function of RNA elements and the molecular mechanisms underlying the RNA-mediated regulation in viral genomes. 

#### 4.1.3. Aptamers Targeting the Structural Elements of the 5′ UTR of the HIV-1 RNA Genome

Reports describing the successful targeting of different functional RNA elements in several viral RNA genomes by inhibitor RNA molecules can be found in the literature. Besides HCV, we have also targeted RNA aptamers against the 5′ UTR of the HIV-1 RNA genome. For this purpose, we developed an innovative approach in which we combined a standard SELEX strategy with an in silico analysis of the selected aptamers, allowing the design of more efficient ones [69].

**Figure 7:**
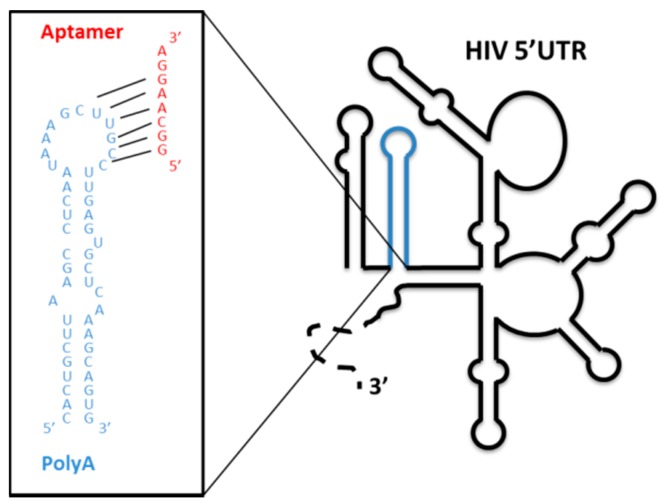
Aptamers selected against the genomic HIV 5′ UTR: A schematic representation of the secondary structure of the 5′ UTR of the viral RNA genome. The well-characterized functional structural elements are depicted. The sequence and secondary structure of the polyA domain is enlarged in the box with blue letters. The octamer consensus sequence of the selected aptamers is indicated in red.

**Figure 8:**
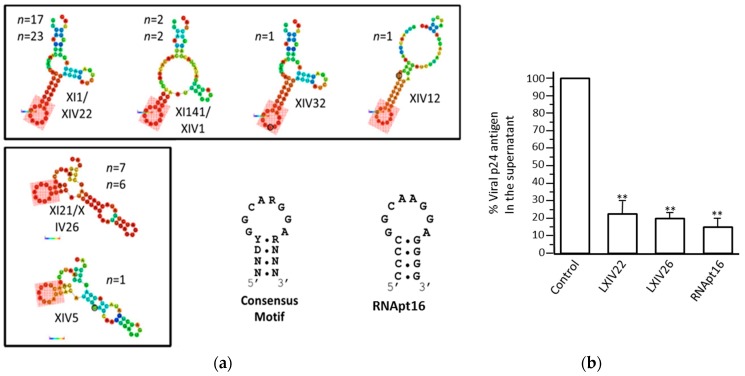
The in silico-designed RNApt16: (**a**) The structural analysis of the representative examples of 64-nt-long selected aptamers shows the minimum free energy isoform. The probabilities of every nucleotide to actually hold the structural conformation shown are represented by a colour code (red, highest; dark blue, lowest). The pink, shadowed box indicates the consensus 16-nt-long structural domain. The sequence and secondary structure of the consensus motif and the designed RNApt16 are indicated at the bottom. R, purine; Y, pirimidine; N, any ribonucleotide, (**b**) HIV-1 inhibition assays: The inhibition of HIV-1 particles production measured as the p24 antigen production by the XIV22, XIV26, and RNApt16 aptamers (compared to a negative control). The data represent the mean of three independent assays. ** The significant differences as compared to the control (*p* < 0.01). The figure is adapted from Reference [69].

The SELEX procedure was applied to a starting population of more than 1 × 10^15^ variants of 64-nt-long RNA molecules. They were binding-challenged against a HIV-1 genomic RNA fragment comprising the first 308 nt of its 5′ UTR. After 11 selection rounds, we observed that the specific 8-nt-long sequence 5′-GGCAAGGA-3′ appeared in high frequency in the RNA population reaching 89.2% from the total analyzed sequences after cycle 14 [69]. Interestingly, this consensus motif is complementary to the sequence exposed in the apical loop of the highly conserved polyA domain of the genomic HIV-1 RNA, indicating that it is the target of the selected aptamers (Figure 7). An in silico structural analysis of the selected RNA molecules containing the consensus octamer sequence reveled the existence of a consensus 16-nt-long structural RNA element, which folded into a stem-loop motif that exposes the octamer sequence in the loop. This 8-nt-long loop was closed by four base pairs of variable sequence (Figure 8a). This result prompted us to hypothesize that the 16-nt structural element was responsible of the aptamer binding capacity and, therefore, its potential inhibitory activity. To validate this hypothesis, an in silico-designed new 16-nt-long RNA aptamer was synthesized. This aptamer was named RNApt16 and consisted in the consensus octamer sequence exposed in a loop closed by a 4-bp-long stem, which the sequence of allows the highest thermodynamic stability of the folded molecule (Figure 8a). 

A comparison study of the inhibition of HIV particles production in the cell culture by the RNApt16 and the two most represented selected aptamers XIV22 and XIV26 demonstrated that the three of them efficiently inhibited viral particle production reaching a maximum inhibition value of 85% for the RNApt16 [69] (Figure 8b). Thus, RNApt16 is the shortest RNA aptamer so far described that efficiently interferes with its corresponding target.

## 5. Conclusions

Viral RNA genomes have developed an information storing system beyond the protein coding one, which stores essential information for the completion of the viral cycle. This system uses structural RNA elements to perform essential functions. The high conservation of these elements among different viral isolates and their important roles in the viral cycle make them potentially good therapeutic targets for new antiviral strategies, which has been confirmed by the reported examples of interfering with their activity by the use of RNA aptamers or other RNA inhibitors. The approach of RNA aptamers targeting highly conserved structural genomic RNA elements offers a potential means of developing new and efficient antiviral strategies. In addition, aptamers are excellent molecular tools to study the functionality of structural RNA domains in viral RNA genomes.
